# Hybrides Coaching in der digitalen Arbeitswelt – Analyse und Ableitung für Forschung und Praxis

**DOI:** 10.1365/s40896-021-00054-4

**Published:** 2021-07-29

**Authors:** Melanie Hasenbein, Jens Kraiss

**Affiliations:** 1 Lehrer-Vogl-Weg 1a, 83623 Dietramszell, Deutschland; 2 Coachline, Obere Seehalde 19, 73660 Urbach, Deutschland

**Keywords:** Coaching, Hybrid, Digital, Neue Arbeitswelt, Co-Creation, Qualitätskriterien, Coaching, Hybrid, Digital, New world of work, Co-creation, Quality criteria

## Abstract

Der vorliegende Beitrag diskutiert die Veränderung von Coaching in der neuen und digitalen Arbeitswelt. Er skizziert dabei hybrides Coaching als vielversprechenden Weg zur Anbindung von Coaching an die hybride Arbeitswelt. Ausgehend von einer Bestandsaufnahme von digitalem Coaching und blended Ansätzen in der Psychotherapie sowie neuen Arbeitsansätzen zeigt er auf, wie hybrides Coaching die Vorteile von direktem face-to-face Coaching und digitalem Coaching vereinen kann. Dabei wird hybrides Coaching als Co-Creation Prozess zwischen Coach und Klient*in beschrieben. Zudem werden die klassischen Qualitätskriterien im Coaching auf hybrides Coaching übertragen und modifiziert. Denn die Sicherstellung von Qualität ist wesentlich, wenn Coaching als professionelles Beratungsinstrument eingesetzt wird. Zudem wird beispielhaft aufgezeigt, wie hybrides Coaching in der Praxis aussehen kann. Schließlich wird herausgearbeitet, welche Forschungsfragen sich daraus ergeben und welche Fragen in der Coachingpraxis zu beantworten sind. Der Beitrag möchte eine theoretische und praktische Diskussion anstoßen, wie hybrides Coaching in der neuen Arbeitswelt gestaltet und durchgeführt wird und wie die Qualität im Coaching sichergestellt werden sollte.

## Abstract (en)

This article focusses on the changes in coaching in the new and digital world of work. It outlines hybrid coaching as a highly promising approach of linking coaching to the hybrid world of work. Based on an inventory of digital coaching and blended approaches in psychotherapy as well as new work approaches, it shows how hybrid coaching can combine the advantages of direct face-to-face coaching and digital coaching. Hybrid coaching is described as a co-creation process between coach and client. The classic criteria of quality in coaching are transferred to hybrid coaching and modified. Ensuring the quality is essential when coaching is used as a professional consultation tool. In addition, examples are shown of what hybrid coaching can look like in practice. Finally, it is worked out which research questions arise from this and which questions have to be answered in coaching practice. The article aims to initiate a theoretical and practical discussion on how hybrid coaching can be designed and implemented in the new world of work and how the quality of coaching should be ensured.

## Einleitung und Problemstellung

In der neuen Arbeitswelt ist der Einsatz von digitalen Medien Normalität geworden. Digitales Coaching, das vor COVID-19 insbesondere in Deutschland eine noch geringe Relevanz hatte, hat nun seinen Platz neben dem klassischen Face-to-Face Coaching erhalten. Sicherlich wird es weiterhin Coaches geben, die diese Form des Coachings nicht präferieren. Auch manche Coachees werden weiterhin den Direktkontakt zum Coach suchen. Die Autoren dieses Beitrags sehen jedoch einerseits mit den Entwicklungen auf praktischer Ebene und andererseits auf Forschungsseite, digitales Coaching als gleichberechtigte Form des Coachings neben dem Präsenzcoaching. Vor allem in der Kombination der beiden Formen von Coaching steckt hohes Potenzial, Coaching auf die neue und digitale Arbeitswelt, in der die Coachees arbeiten, anzupassen. Die Kundenbedürfnisse der Coachees haben sich durch branchenspezifische Transformationen und die veränderte Zusammenarbeit (z. B. verteilte Teams, selbstorganisierte Teams, autonomes Arbeiten) weiterentwickelt und das wirkt sich auch auf Coaching aus. Diesem soll in dem vorliegenden Artikel nachgegangen werden. Dabei wird zum einen die Frage aufgeworfen, welche Anforderungen die neue Arbeitswelt an Coaching stellt und welche Möglichkeiten bereits digitales Coaching bietet. Zum anderen wird analysiert, welche Ansatzpunkte die Blended Therapie liefert, die dem Coaching bereits einige Schritte voraus ist. Auf Basis dessen soll die Frage beantwortet werden, was sich davon auf einen neuen hybriden Coachingansatz übertragen lässt und wie dies in der Umsetzung aussehen kann. Damit eine solche neue Form des Coachings den Status eines professionellen Beratungsinstruments erfüllt, wird zudem die Frage gestellt, wie die Qualität bei einem hybriden Coachingansatz sichergestellt werden kann. Daraus ergeben sich wiederum Ableitungen für die Coachingpraxis und -forschung.

## Neue und hybride Arbeitswelt

Die Art und Weise des Arbeitens hat sich in der sogenannten VUCA-Welt (Volatility, Uncertainty, Complexity, Ambiguity) und mit COVID-19 verändert und wird sicherlich einen weiteren Wandel erfahren. Dies hat Auswirkungen auf die Arbeitsgestaltung. Wir arbeiten vermehrt digital und flexibler zusammen. Nach COVID-19 werden einige Menschen gerne wieder an ihren Arbeitsplatz zurückgehen, andere werden Gefallen daran gefunden haben, von zuhause zu arbeiten. Weil sie beispielsweise keine Zeit im Berufsverkehr verlieren, dadurch weniger gestresst und produktiver arbeiten können. Manche möchten ganz flexibel entscheiden können, ob sie heute oder morgen von zuhause oder im Büro arbeiten. Damit können Berufs- und Privatleben mehr in Einklang gebracht werden. Diese Form des Arbeitens kann als hybrides Arbeitsmodell beschrieben werden. Sie umfasst eine Kombination aus mobilem Arbeiten und halbmobilen Arbeiten sowie bürobasiertem Arbeiten. Der Begriff hybrides Arbeiten ist auch dadurch gekennzeichnet, dass die Art und Weise oder die Form der Zusammenarbeit sich verändert. Nicht nur die Arbeitsperson selbst hat verschiedene Möglichkeiten, die Form der Arbeit zu gestalten, auch zwischen den Arbeitspersonen entstehen Mischformen der Zusammenarbeit. So arbeitet eine Person im Homeoffice und die andere Person im Büro der Arbeitsstätte. Das ist vergleichbar mit dem hybriden Antrieb eines Fahrzeuges, bei dem agil zwischen einem Elektromotor und Verbrennungsmotor gewechselt werden kann. Analog zu diesen veränderten Kundenbedürfnissen haben sich die Mitarbeiterbedürfnisse in der hybriden Arbeitswelt maßgeblich verändert. Hinzu kommt eine neue Haltung gegenüber Arbeit und Arbeiten, der sogenannten Arbeit 4.0. In diesem Zusammenhang fällt häufig der Begriff „New Work“. „*Die neue Perspektive von Arbeit geht weg von einer reinen Austauschbeziehung, z.* *B. Geld für Arbeitsleistung, hin zu einer sinnerfüllenden Tätigkeit. Auch das ist nichts Neues und diese Entwicklungen haben wir bereits schon gesehen. Sie bekommen jedoch mit den aktuellen Bewegungen eine stärkere Relevanz“* (vgl. Hasenbein 2020, S. 16).

Was bedeutet das? Schermuly (2016) hat dies in seinem Buch „New Work – gute Arbeit gestalten“ in Anlehnung an Spreitzer (1995, 2008) herausgearbeitet. Er beschreibt vier Komponenten, die neue, gute Arbeit ausmachen. Diese umfassen das Erleben von Kompetenz, Bedeutsamkeit, Selbstbestimmung und Einfluss. Dahinter steht das Konzept des psychologischen Empowerment, das das individuelle Erleben des Menschen im Arbeitskontext in den Fokus stellt. Dies beinhaltet ein positives Kompetenzgefühl, dass das Individuum den täglichen Anforderungen bei der Arbeit mit seinen Kompetenzen begegnen kann. Kompetenzen: Dies sind fachliche, soziale, methodische und personale Kompetenzen, um ein kohärentes Kompetenzgefühl empfinden zu können. Bedeutsamkeit kann auf verschiedenen Ebenen wahrgenommen werden. Die eigene Arbeit wird vom Individuum und von der Organisation als wichtig betrachtet, die Arbeit wird als bedeutsam für das eigene Leben bewertet, die Arbeit wird als wertvoll für andere sowie als Beitrag für die Gesellschaft empfunden (vgl. Hasenbein 2020). Selbstbestimmung umfasst das Erleben von Autonomie und Freiheit bei der Ausführung der Arbeit. Einfluss zu haben, bedeutet, die eigene Arbeit so wahrzunehmen, dass diese die Resultate im jeweiligen Arbeitsumfeld positiv beeinflussen kann. Das Zusammenspiel dieser vier Aspekte führt, so konnte es auch eine Metastudie zeigen, zu einem Gefühl von psychologischem Empowerment im eigenen Arbeitsumfeld (Seibert et al. 2011; Spreitzer 2008). Um dieses Empowerment bzw. Self-Empowerment bei Führungskräften und Mitarbeitern zu unterstützen, braucht es entsprechende Ansätze. Hier kommt Coaching ins Spiel. Gerade für Führungskräfte ist in der heutigen digitalen und agilen Zeit, die zunehmend Selbstorganisation und Selbstregulation erfordert, Selbstführung von zentraler Bedeutung. Das setzt voraus, sich selbst gut zu kennen und einschätzen zu können. Gerdenitsch und Korunka (2019) drücken dies sehr trefflich aus: *„Selbstführung beinhaltet verschiedene verhaltensbezogene, kognitive und emotionale Strategien wie beispielsweise das Setzen eigener Ziele, die Selbstbeobachtung, das Visualisieren der eigenen Leistung oder die Reflexion über die eigenen Annahmen“* (S. 181). Die Selbstreflexion, die durch Coaching angestoßen und gefördert werden soll, spielt hierbei eine zentrale Rolle. Breidenbach und Rollow (2019) sprechen in diesem Zusammenhang von „Inner Work“, das in Zeiten der „New Work“ Transformation bei Führungskräften und Mitarbeitern:innen gefordert ist. Coaching kann diesen individuellen und persönlichen Entwicklungsprozess der „inneren Arbeit“ professionell begleiten. Dies kann durch einen Coach in Persona, mit Hilfe von digitalen Coachingprogrammen oder in Kombination erfolgen.

Was zu der neuen Art des Arbeitens und Zusammenarbeitens hinzukommt, ist eine wachsende Flexibilisierung der Arbeit auf verschiedenen Ebenen. Dies zeigt sich durch eine zunehmende Vernetzung innerhalb von Organisationen – über Bereichs- und Hierarchiegrenzen hinweg – und zwischen Organisationen. Zudem verändern sich die Arbeitsbeziehungen, z. B. durch die virtuelle Zusammenarbeit von Teams, die Arbeit gemeinsam mit Robotern und über flachere Hierarchien bis hin zu selbstorganisierten und autonomen Teams. Weiterhin wandeln sich die Möglichkeiten des Arbeitens, was Zeit und Ort betrifft. Zunehmend gibt es eine Kombination aus Zusammenarbeiten im direkten und virtuellen Kontakt oder beides gleichzeitig. Mehrere Kollegen:innen sind im Büro und ein einzelne sind per Videokonferenz zugeschaltet. Möglicherweise kann ich sogar wählen, was aktuell besser in meinen Arbeits- und Lebenskontext passt. Solche hybriden Formen der Zusammenarbeit wird es sicherlich zukünftig zunehmend und selbstverständlicher geben.

## Coaching in der hybriden Arbeitswelt

Die Auswirkungen einer hybriden Arbeitswelt werden sich auch und gerade im Coaching zeigen. Dabei muss Coaching als eigene Profession eine Perspektive gegeben werden und in dem Kontext von Digitalisierung und Hybridisierung selbst zu einer Professionsbildung beitragen. Dies kann entsprechend durch ein professionelles Ausbildungsangebot erfolgen, das die Qualitätskriterien von Coaching erfüllt. Hier sollten alle Vertreter des professionellen Coachings selbstbewusst die Chance sehen und nutzen. Es wäre bedauerlich, wenn Coaching als Methode in einem spezifischen Beratungsformat ggf. seine Bedeutung verliert (DBVC 2019). Es gibt zwar für Coaching keine eigene, historisch etablierte Wissenschaft, wohl aber eine fundierte Wissenschaftsorientierung.

Wir werden uns zukünftig damit auseinandersetzen müssen, wie wir die Professionalität des Coachings der hybriden Arbeitswelt anpassen oder besser, wie wir diese neue Arbeitswelt mitgestalten. Dies kann beispielsweise als Coach im Führungsalltag von Managern:innen erfolgen. Coaching muss dabei der Flexibilisierung der Arbeitswelt gerecht werden und vor allem beweglicher, agiler sein und damit einem hybriden Ansatz folgen. Dieser neue hybride Coachingansatz ist geprägt durch eine hohe Beweglichkeit und Anpassung an die aktuelle Situation eines Coachee. Die Kundenbedürfnisse oder Customer Centricity rücken dabei besonders in den Mittelpunkt und gestalten das Coaching. Bis dato wird sehr häufig in der Wissenschaft rein unterschieden zwischen Präsenzcoaching und digitalem Coaching. Die Verbindung des Coachingprozesses zu einem Blended-Coaching ist in der Forschung aktuell wenig bis gar nicht beleuchtet. Vor allem die Qualitätssicherung und die Wirksamkeit beider Formate ist bisher nicht ausreichend untersucht. Im Alltag nutzen Coaches jedoch bereits diese Form der Durchführung. Die Flexibilisierung der Arbeitswelt und damit einhergehend die Entstehung eines hybriden Coachingansatzes stellt besondere Anforderungen an die Professionalität von Coaching. Dabei können die Praxis und Forschungsergebnisse aus dem digitalen Coaching und der Blended-Psychotherapie sehr gute Anhaltspunkte liefern.

## Status Quo und Trends im digitalen Coaching

Digitales Coaching ist im deutschsprachigen Markt angekommen. COVID hat die Digitalisierung von Coaching beschleunigt und geradezu einen Boom der Anerkennung freigesetzt. Was im internationale Kontext schon lange Realität und Normalität ist, hat sich hierzulande mit COVID etabliert. Die Rauen Coaching-Marktanalyse (2020) (vor COVID) hat noch offenbart, dass nur 7,7 % der deutschen Coaches mit Videokonferenzen arbeiten. Interessant ist dabei, dass in der international angelegten ICF Global Study (2019) bereits 48 % der weltweit befragten Coaches regelmäßig Audio/-Videoplattformen einsetzen. Seit Beginn der Pandemie hat sich das Bild jedoch deutlich verändert. In West-Europa hat laut der ICF Global COVID Study (2020) mit über 10.000 Teilnehmern:innen digitales Coaching um 81 % zugenommen. Das zeigt, dass bei einer Notwendigkeit digitales Coaching genutzt wird. 67 % der Coaches aus West-Europa lehnen es ab, nach der Pandemie wieder ausschließlich zu den Methoden zurückzukehren, die sie vor der Pandemie genutzt haben. Weiter sind 86 % der Teilnehmer:innen aus West-Europa bereit, neue Technologien für ihre Coachingsessions zu nutzen, so die befragten Coaches der ICF Covid Studie. In einer Studie zum Einsatz von digitalen Medien gaben auch 82 % an, Videotelefonie eignet sich als digitales Medium und 85 % sehen die hohe Flexibilität beim Einsatz von digitalen Medien als positiv (Sakowski und Ahrens 2020). Digitales Coaching, Therapie oder Beratung entfalten dabei genauso ihre Wirkung wie Präsenzformate (Baumeister et al. 2018; Berninger-Schäfer 2018; Geißler et al. 2013). Vorausgesetzt jedoch, dass das Coaching in seiner Gesamtheit auch auf ein digitales Coaching angepasst wird. Die veränderten Rahmenbedingungen und die Qualitätskriterien müssen im digitalen Coaching modifiziert werden. Denn eine sehr wirksame Präsenzmethodik funktioniert nicht zwangsläufig genauso im digitalen Coaching. Wichtig ist, genau auf Anliegen, Zielsetzung, Zielgruppe, Präferenzen und Bedürfnisse zu schauen. Studien zu digitalem Coaching werden weiter intensiviert. Beispielhaft sei die laufende Studie der Universität Zürich (Allemand et al. 2020) in Zusammenarbeit mit Wia Ventures genannt. MindHike ist eine digitale Coachingapplikation und unterstützt die Fähigkeit zur Selbstkontrolle zu verbessern. Die Studie hat Ende 2020 begonnen.

Zentral und oftmals vernachlässigt ist in Studien oder Umfragen die Coacheeperspektive. Was möchte der Coachee, ein digitales Coaching oder ein Coaching in Präsenz oder beides? Welche Bedürfnisse bestehen beim Coachee? Hier ist ein klarer Trend zu digitalem Coaching zu sehen. Das liegt schon daran, dass sich die Lernpräferenzen der Generationen, die nun in Management-Funktionen sind oder kommen, deutlich verändern. Das wirkt sich auf den Coachingmarkt aus. Die erwähnte Coacheeperspektive und das neue digitale Lernverhalten von Coachees darf dabei in den viel geführten Diskussionen nicht vernachlässigt werden. So haben Bachmann et al. (2019) bei einer vergleichenden Analyse des Nutzungsverhaltens digitaler Medien im Coaching identifiziert, dass Personalverantwortliche der Digitalisierung im Coaching offener gegenüberstehen als externe Coaches. Auch in der Delphi-Studie über die Zukunft des Coachings von Schermuly (2020) zeigen sich bei bestimmten Trends, dass Auftraggeber:innen von Coaching digitalisierte Szenarien wahrscheinlicher einschätzen als die Coaches selbst.

In Ergänzung sei erwähnt, dass die Evidenzlage bei digitaler Psychotherapie eindeutiger ist. Digitale Psychotherapie ist wirksam (Ebert 2019; Baumeister et al. 2018; Berninger-Schäfer 2018). Hier gibt es Meta-Studien (Andrews et al. 2018; Wagner et al. 2014), die auch mit einer hohen Anzahl von Probande:innen eine aussagefähige Datenbasis liefern. Es gilt festzuhalten, dass die Forschung zur Wirkung von digitaler Psychotherapie deutlich weiter ist als zu digitalem Coaching und valide Ergebnisse liefert. Dies liegt sicherlich daran, dass die Psychologie, respektive die Psychotherapie, in der Wissenschaft als eigene Disziplin bereits lange Zeit verankert und damit eine wissenschaftlich fundierte Studienlage möglich ist. Das hat dazu geführt, dass das Bundesinstitut für Arzneimittel und Medizinprodukte (BfArM), digitale Gesundheitsanwendungen nach vorgegeben Standards bewertet und in einem transparenten Prozess in ein Verzeichnis (DIGA-Verzeichnis) aufgenommen hat. In der Coachingbranche fehlt es dazu an klaren Qualitätskriterien, die von einer staatlichen Institution vorgegeben werden und an denen sich der Markt orientieren kann. Das führt dazu, dass der digitale Coachingmarkt unübersichtlich bleibt und Qualität für die Coachees zu wenig nachvollziehbar ist.

Die Business Coachingbranche befindet sich in einer disruptiven Phase. Dieser radikale Umbruch hat sich bereits vor mehreren Jahren abgezeichnet. Dabei sind wesentliche Veränderungen zu erkennen, auf die sich die Coachingbranche und vor allem jeder einzelne Coach einstellen sollte und seinen Weg für sich finden muss. Das fällt nicht jedem leicht. Manche Coaches, die über Jahrzehnte ausschließlich in Präsenz gecoacht haben, sehen möglicherweise nicht den Mehrwert von digitalem Coaching. Für einige ist es oder war es vor COVID ein absolut unbekanntes Terrain. Das ist entsprechend mit Unsicherheiten verbunden. Hier gilt es, dies in Aus- und Weiterbildungen für Coaches zu berücksichtigen. Coaching wird zudem nicht mehr als persönliche Entwicklungsmaßnahme gesehen, die nur Wenigen zugänglich ist. Coaching ist ein Geschäftsmodell geworden, das seine komplette Verbreitung in den Personalentwicklungsabteilungen von Unternehmen und darüber hinaus gefunden hat. Die digitalen Coachingplattformen bieten darüber hinaus Coaching effizient, skalierbar und für alle an. Die Entwicklungen zum Coaching 4.0 verändern den Markt zudem durch diverse digitale Dienst- und Vermittlungsleistungen wie bettercoach, Sharpist, CoachHub und Haufe Coaching (vgl. Middendorf und Ritter 2020). Dabei wird das Management des Coachings an sich (Coach Pool, Prozesse) als auch die Durchführung des Coachings ausgelagert zu externen Anbietern. Im Zuge der beschriebenen Geschäftsmodell-Veränderungen hat sich die Segmentierung von Coaching verändert. Drei unterschiedliche Segmente sind aktuell erkennbar.

1. Selbstcoaching: Das sind Menschen, die digitale Coachingangebote nutzen, die zur Verfügung stehen, ohne dass es einer persönlichen, direkten Begleitung bedarf. Das sind Videos, APPs, Bots, Tutorials, Bücher, etc. Der Markt boomt, die Angebote sind unübersichtlich und die Qualität schwierig überprüfbar. Man kann hierbei auch von „Selbstoptimierungs-Coaching“ sprechen. Diese Form des Coachings setzt auf schnelle und bequeme Hilfe und versucht eine Veränderung durch Selbststudium herbei zu führen. Jemand, der die Kraft des Dialogs schätzt, wird das Angebot eher nicht nutzen (Wrede und Zimmermann 2020).
2. Standardthemen: Diese Themen werden hauptsächlich von den digitalen Coachingplattformen in Zukunft bedient. In der Regel aus einer Kombination von direktem, persönlichem Kontakt zu einem Coach und in Kombination mit standardisierten, themenspezifischen, digitalen Modulen sowie Selbstcoachinganteilen. Im Fokus steht die Skalierbarkeit. D. h. es werden Module geschaffen, die in der Regel eine hohe Anzahl von Coachingstandardthemen abdecken und vielen Menschen zur Verfügung gestellt werden. Einmal erstellt, können die Module unbegrenzt genutzt und verkauft werden. Zielgruppen sind hier Fachkräfte, Experten und Personen bis zum mittleren Management
3. Komplexe Anliegen: Die komplexen Fragstellungen sind sehr individuell, tiefgründig und in der Ausprägung facettenreich. In der Regel ist die Zielgruppe hier das TOP Management. Das ist eher das Segment des hochpreisigen Coachings. Das Segment wird dann in der Regel von Präsenzangeboten in Ergänzung von digitalen, direkten Kommunikationsmöglichkeiten (z. B. Videoplattformen, VR-Angebote, Messenger) bedient.

Coaches sind Experten:innen für Veränderungsprozesse. Gerade in digitalen Transformationsprozessen in Unternehmen werden sie zukünftig mit Beratern in interdisziplinären Teams diese Transformationen begleiten. Dazu werden jedoch Kompetenzen aus der Organisationsentwicklung erforderlich sein (Schermuly 2020). Digitales Coaching und besonders die Formate, Methoden, Tools werden dann in Zusammenarbeit mit dem digitalen Berater:in genutzt. Coaching wird in Zukunft deutlich digitaler. KI-(Künstliche Intelligenz)gestützte Tools (z. B. Bots), VR-Anwendungen, Avatar-gesteuerte Sessions, Algorithmus-basierte Programme (z. B. zur Coach Auswahl) sind bereits Realität. KI, die auf emotionale Körperreaktionen in der Kommunikation reagieren, sind in der Entwicklung und liefern bereits gute Ergebnisse. So haben Entwickler:innen des Institute For Creative Technologies (ITC) bereits vor Jahren damit begonnen emotionale Intelligenz in KI-Lösungen zu integrieren. Das Ergebnis sind „empathische“, virtuelle Agenten:innen, die menschliches Verhalten lesen, verstehen und darauf antworten können. Ein Beispiel ist Avatar „Ellie“, die Militärangehörige nach ihrem Einsatz bei „post-traumatic stress disorder (PTSD)“ begleitet. Ellie kann Gestik, Körperhaltungen, Gesichtsausdrücke wahrnehmen und darauf in ihren Antworten reagieren (Cremin 2016).

Qualitativ hochwertiges Coaching ist jedoch ein iterativer Prozess und gewinnt an Mehrwert durch eine Art Co-Creation zwischen Coachee und Coach. Diese individuelle, personenbezogene Begleitung, die ein realer Coach abdeckt, ist die große Herausforderung für KI Coaching. KI Coaching hat großes Potenzial, den Coachingprozess langfristig maßgeblich zu beeinflussen. Gerade in den logischen, nachvollziehbaren Coachingschritten wie Zieldefinition, Diagnostiktools (z. B. Persönlichkeitstests), Implementierung von Zielen im Alltag, sowie Monitoring oder Tracking ist KI geeignet. Die größten Herausforderungen von KI Coaching liegen dabei in der Problemidentifizierung zu Beginn, in reflexiven Prozessen und bei Zieländerungen während des Coachingprozesses. Dann sind aktuell reale Coaches gefragt, die Reflexion zulassen und steuern. Zudem könnte KI Coaching bei sich ergebenden ethischen Fragestellungen problematisch werden und sollte unbedingt durch die Entwickler:innen beachtet werden. KI Coaching ist daher als Partner zu verstehen. Die Kombination der Benefits aus realen Coaches und KI Coaching ist dabei ein Weg für weiterhin qualitativ hochwertiges Coaching (Schermuly und Graßmann 2020). Digitale Coachinganbieter entwickeln sich oftmals nicht aus der Coachingbranche heraus, sondern aus der IT-Branche. Damit stellt sich insgesamt die Frage, will man branchenfremden IT-Experten:innen oder Finanzinvestoren:innen die Entwicklung von Coaching APPs überlassen (Schermuly 2020).

Die neue digitale Generation der Führungskräfte und damit der Coachees für Coaching ist in den Chefetagen angekommen. Diese haben deutlich veränderte Lernpräferenzen und ein neues Lernverhalten. Coaching muss sich dem anpassen. Coaching wird sich in Zukunft mehr an den Coacheebedürfnissen orientieren. Damit geht der Trend weg von Produktorientierung hin zu Kundenorientierung. Das Lernverhalten und damit auch die Lernkultur von Organisationen oder Personen hat sich in den letzten Jahren deutlich verändert. Eine Mischung aus „Self-Organised-Learning“, „Peer-Learning“ und „Direct Learning“ hat sich durchgesetzt. Im Coaching wird sich dies dahingehend auswirken, dass Coachees auch alle drei Lernformen nutzen möchten. Blended-Learning als gemischter Ansatz gilt bereits im Corporate-Learning als nachhaltige Lernform. Dies können verschiedene Studien und Trendanalysen belegen (mmb Institut 2019, 2020; Schmid et al. 2018; KOFA 2019). Dabei werden Elemente des digitalen Lernens und Präsenzformate sinnvoll miteinander verknüpft. Dies gilt es auch auf das Coaching zu übertragen. Manche Studien (vgl. Thalheimer 2017) zeigen sogar, dass Blended Learning dem reinen Classroom-Lernen in vielen Einflussfaktoren deutlich überlegen ist. Die Meta-Analyse von Thalheimer sieht das insbesondere daran begründet, dass die Kombination aus stark wirksamen Lernmethoden eingesetzt wird. Blended-Learning erzielt dabei vor allem bei den Faktoren Wirkung der Maßnahme, Flexibilität im Lernen und Produktivität ein besseres Ergebnis (Stein und Graham 2020). Wichtig ist, dass nicht das Lernformat (digitales Learning vs. Classroom) für eine Wirkung entscheidend ist, sondern die Lernmethode dahinter so Thalheimer (2017).

Die Ausführungen zum Status Quo im digitalen Coaching und die Darlegung der wesentlichen Trends im Coaching zeigen eine disruptive Veränderung. Ein klarer Weg ist noch nicht eindeutig erkennbar.

Für einen klaren Weg bedarf es der Unterstützung aus der Wissenschaft. Die Etablierung von Studiengängen zu Coaching ist für die Professionalisierung von Coaching dabei sehr wichtig. Damit könnte es gelingen, wissenschaftlich fundierte Ableitungen zu erhalten. Es wird auf jeden Fall deutlich digitaler und komplexer im Coaching zugehen. Das bedeutet eine große Veränderung für alle Coaches. David Goldsmith (2019) hat in seinem Beitrag im Rahmen der Coacharya „The Future of Coaching“, Coaches Folgendes empfohlen: „1. Be a true professional, 2. become a learning expert, 3. solve the hardest problems, 4. reinvest yourself, 5. use robots, 6. be a role model, 7. do what a BOT cannot“.

## Status Quo Blended Therapie

**Figure 1:**
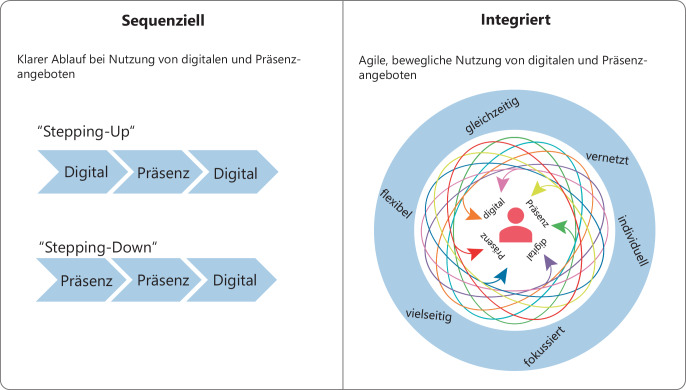

Die breite Evidenzlage zur Effektivität von internetbasierten therapeutischen Interventionen in den verschiedensten Disziplinen wie Angststörungen, Depression, PTSD etc. führt seit einiger Zeit dazu „das Beste aus beiden Welten“, digital und Präsenz, miteinander zu verknüpfen. Unter Blended-Psychotherapie versteht man die verzahnte „Nutzung etablierter Präsenz-Therapieansätze mit dem Einsatz elektronischer Medien“ (Baumeister et al. 2018). Erbe et al. (2017) unterscheiden dabei zwischen zwei Formen der Blended-Psychotherapie, die in Abb. 1 aufgeführt sind: *sequentiell* und *integriert*. Im Bereich der sequenziellen Form gibt es zwei Varianten. 1. Die Stepping-Up Variante. Dabei gehen die Digitalinterventionen den Präsenzinterventionen voraus. Diese Variante wird oftmals zur Überbrückung von Wartezeiten genutzt. Die zweite Variante, Stepping-Down, liegt dann vor, wenn die Präsenzintervention einer Digitalintervention vorausgehen. Dies wird insbesondere zur unterstützenden Langzeittherapie oder im Bereich der Nachsorge (aftercare) eines stationären Aufenthaltes genutzt. Die Stepping-Care Ansätze dienen insbesondere dazu, die Effektivität und Effizienz einer Therapie zu steuern. Führen beispielsweise die Digitalangebote nicht zu einer Reduktion der Symptome, kann auf eine kostenintensivere Präsenzbehandlung gewechselt werden. Der sequenzielle Ansatz ist zwar starrer in seiner Anordnung, bietet für den Patienten:innen jedoch einen eindeutigen Ablauf und ist für beide Seiten gut steuerbar. Bei der integrierten Form der Blended-Psychotherapie geht es um die gleichzeitige Nutzung von Präsenz- und Digitalinterventionen. Die Interventionen werden hierbei untereinander vernetzt. Damit tragen beide Arten substanziell zum Erfolg einer Therapie bei. Im integrierten Einsatz kann damit eine Präsenztherapie in die Digitaltherapie zur weiteren Qualitätskontrolle oder der Krisenintervention integriert werden. Oftmals geht es im integrierten Ansatz, um die Flexibilisierung der Psychotherapiemöglichkeiten (Baumeister et al. 2018). Die Varianten bei der integrierten Form sind vielseitig und es lässt sich damit während der Therapie der Fokus auf die Steigerung der Wirksamkeit oder der besseren Ressourcennutzung legen. Der integrierte Ansatz ist insgesamt viel beweglicher, agiler und kann damit besser auf die Patientenbedürfnisse angepasst werden. Das setzt jedoch voraus, dass die Patienten:innen gut abgeholt und nicht überfordert werden. Im internationalen Kontext gibt es Studien, die den Einsatz von Blended Care untersucht haben (Kemmeren et al. 2016; Titzler et al. 2019; Wentzel et al. 2016). In Deutschland wird das Projekt PsyTom zur weiteren Untersuchung von Blended-Psychotherapie eingesetzt (Knaevelsrud, Böttcher & Pohl 2019). PsyTOM wird durch den Innovationsfonds des Gemeinsamen Bundesausschusses gefördert und soll 2021 starten. Ziel des Projekts ist die Untersuchung einer verfahrens- und störungsübergreifenden Integration von Blended-Care in die Routineversorgung. Die dabei entwickelten digitalen Module sollen später allen gesetzlich Versicherten zur Verfügung stehen. Aus Blended-Therapie-Studien können folgende Kernaussagen abgeleitet werden, deren Umsetzung und Wirksamkeit auch in einem blended, respektive hybriden Coaching zu überprüfen wären:

- Die Wirkung einer stationären Behandlung wird mit Unterstützung von Digitaltherapie deutlich verbessert.
- Die Unterstützung einer Präsenz-Langzeittherapie durch Digitalangebote reduziert deutlich die Symptome.
- Mit Blended Psychotherapie werden Rückfall- und Abbruchraten von Präsenzangeboten deutlich reduziert.
- Bei Start mit Digitalangeboten wird das Annehmen einer Präsenzpsychotherapie deutlich verbessert und für den Einsatz während einer Warteliste bietet sich zum Start zunächst Digitaltherapie an.
- Blended-Psychotherapie erhöht deutlich die Flexibilität bei der Gestaltung von Therapiemaßnahmen. So lässt sich in einem Blended-Ansatz eine Therapie besser in den Alltag integrieren und an die individuellen Bedürfnisse anpassen.
- Digital und Präsenzelemente müssen miteinander in Beziehung stehen und nicht in zwei separaten Behandlungssträngen agieren.
- Die Anzahl von Digital- oder Präsenzangeboten sollte gut zu den Bedürfnissen des Patienten:in passen.

## Hybrides Coaching

Die Passung zum Coachee hat im Coaching aus Sicht der Autoren dieses Beitrages eine ganz zentrale Bedeutung. Deshalb soll an dieser Stelle auch von hybridem Coaching gesprochen werden, das die passende Brücke von digitalem und analogen Coaching je nach Coacheebedürfnissen schafft. Blended-Ansätze haben in der Vergangenheit den Fokus primär auf eine Kombination von Offline- und Digitalangeboten gelegt. Hybrid jedoch hat eine weitergehende Bedeutung. Es handelt sich hierbei um eine Co-Creation zwischen Coach und Coachee, bei der Coach und Coachee flexibel entscheiden können, wann sie ihre Coachingsessions in Präsenz und wann digital durchführen und wie viele Sessions sie in digitaler oder analoger Form gestalten möchten. Der Begriff Co-Creation wurde geprägt durch Prahalad und Ramaswamy (2000) in ihrem Artikel „Co-opting Customer Competence“. Dabei haben sie beschrieben wie der Kunde:in von der Publikumsrolle bei der Produktentwicklung in eine aktive Rolle auf der Bühne geht. Die Möglichkeiten des Internets sahen sie als grundlegend für diese Veränderung. Der Kunde:in wird hierbei zu einer neuen Quelle an Kompetenz für ein Unternehmen. In dieser Funktion bringen sie Wissen und Fähigkeiten mit und ihre Bereitschaft, zu lernen und zu experimentieren sowie die Fähigkeit einen Dialog aktiv zu führen. Unter dem Aspekt „encouraging customers“ beschreiben Prahalad und Ramaswamy (2000) einen Dialog auf Augenhöhe zwischen Kunde:in und Unternehmen. Eine Art gemeinsamer Schöpfungsprozess, der unerlässlich für den Zweck, die Bedeutung und die Qualität eines Dialogs mit dem Kunden:innen ist. Der Kunde:in wird in diesem Dialog zu einem „Cocreator“ der Inhalte seiner zukünftigen eigenen „customer experience“. In diesem Entwicklungsprozess ist es sehr wichtig, mit dem Kunden:in aktiv, explizit und permanent in den Dialog zu gehen und dies erfordert eine hohe Flexibilität. Die Co-Creation im hybriden Coaching beschreibt genau den Ansatz von Prahalad und Ramasway. Es geht darum, den Coachee als eine Quelle an Kompetenz zu sehen und ihm Wahlmöglichkeiten und eine hohe Flexibilität für seine Bedürfnisse im hybriden Coaching anzubieten. Im hybriden Coaching ist für eine hohe Qualität im Coaching der aktive, explizite und permanente Dialog so wichtig. Genau diese Art des Dialogs lässt sich so gut in der Verzahnung von Präsenz und digital führen. Als Beispiel für die Aufrechterhaltung eines permanenten Dialogs ist die Nutzung eines Messenger Dienstes. Co-Creation im hybriden Prozess ermöglicht eine besondere Form des Prozessmanagements bei hoher Komplexität und Mehrdeutigkeit. Der hybride Prozess ist komplex in den Anforderungen für den Coachee als auch und ganz besonders für den Coach. Die Coachingmöglichkeiten aus Präsenz und digital in Verknüpfung mit dem Anliegen und der Arbeitswelt des Coachees erfordern daher diese Co-Creation. Das hybride Coaching geht in seinem Grundmodell in eine Coacheeorientierung und weg von einer Produktorientierung im Coaching. Dabei ist immer die Frage zu stellen, was passt aktuell am besten zu den Bedürfnissen des Coachees und den Coachingerfordernissen. Diese Form des integrierten Ansatzes ermöglicht die Verzahnung von klassischem Präsenzcoaching mit verschiedenen digitalen Medien und Tools wie z. B. mit Videokonferenzsystemen, mit Coaching-Apps, Avataren und Virtual Reality. Je nach Bedarf und Zielsetzung können diese individuell zusammengestellt und eingesetzt oder auch gleichzeitig genutzt werden. Die Blended-Therapie öffnet dabei dem hybriden Coaching den Weg durch das Grundmodell eines integrierten Ansatzes. Dieser Ansatz berücksichtigt und fokussiert auf die Coacheebedürfnisse im Coaching. Er bietet die notwendige Flexibilisierung von Coachingmöglichkeiten. Die Ergänzung des integrierten Ansatzes um die Form der Co-Creation geht noch einen Schritt weiter und gibt Coaches die Chance, die Coacheebedürfnisse konkret mit ins Coaching einzubinden. Vor allem kennen die Coachees diese Form der Zusammenarbeit aus ihrer hybriden Arbeits- und Lernwelt. Das scheint ein Mehrwert, den hybriden Coachingansatz auf dieses Fundament zu stellen. Gerade in der zukünftigen hybriden und flexibilisierten Arbeitswelt gleicht sich das Coaching mit der hybriden Form in seiner Vorgehensweise an. Damit bedient der hybride Ansatz nicht nur die Coacheebedürfnisse, sondern auch die Zukunft des Coachings in seiner Gesamtheit. Das ist eine große Chance für das Coaching, die in vielen Branchen stattfindende Transformation zukunftsorientiert zu begleiten. Weiter nehmen wir für den hybriden Coachingansatz mit, dass beim Design von hybriden Coachingmaßnahmen ganz entscheidend die Coachingmethode sein sollte. Es sollte die Methode gewählt werden, die je nach Format (digital – Präsenz) die beste Wirkung erzielt. Dies besser zu erforschen und zu verstehen sollte ein Fokus von Coaching sein. Coacheeorientierung heißt auch Qualitätssicherung und kontinuierliche Verbesserung. Und für einen professionellen Ansatz braucht es Qualitätskriterien, die im Folgenden anhand der klassischen Qualitätskriterien im Coaching überprüft und für hybrides Coaching modifiziert werden.

## Qualitätskriterien im hybriden Coachingansatz

Damit ein hybrider Coachingansatz nicht willkürlich wirkt und wie Coaching generell den Status eines professionellen Beratungs- und Personalentwicklungsinstruments erhält, ist es unumgänglich, dass diese Form des Coachings überprüfbaren Qualitätskriterien standhält. Hess und Roth (2001) differenzieren hierbei in Struktur‑, Prozess- und Ergebnisqualität:

- Strukturqualität: Hierunter fallen die Rahmenbedingungen von Coaching. Das sind das Setting von Coachings, Aus- und Weiterbildung für Coaching, und Anforderungen an das Coaching sowie Kompetenzen des Coaches.
- Prozessqualität: Hierunter fallen alle prozessualen Aspekte von Coaching. Dazu gehört die Diagnostik/Analyse, die Methodik/Didaktik und Interventionen im Coaching sowie die Prozessgestaltung und -steuerung. Weitere wichtige Aspekte sind die Vertragsgestaltung, die Dokumentation sowie die Supervision und kollegiale Beratung.
- Ergebnisqualität: Hier steht das Ergebnis von Coaching im Fokus. Dazu gehört die Zielerreichung im Coaching, der Transfer von Coachingergebnissen und Feedback des Coachees sowie eine Evaluation.

**Figure 2:**
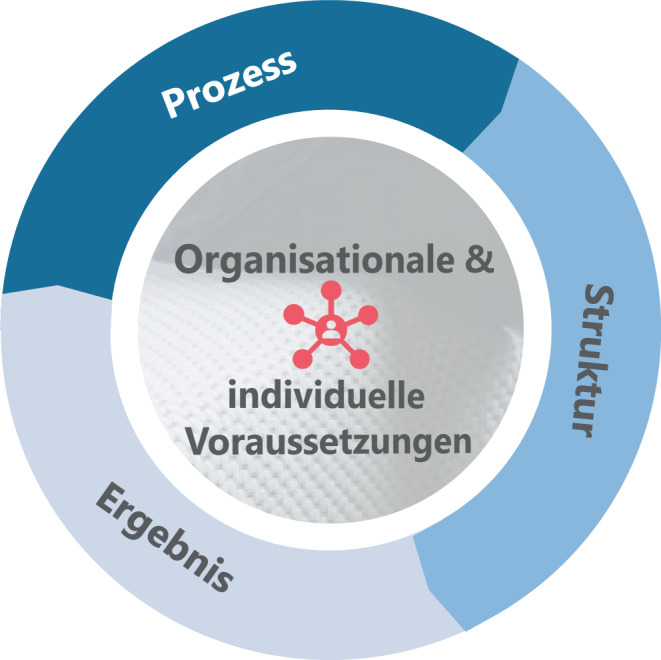

Ergänzt werden diese Kriterien aus Sicht der Autoren um die individuellen Voraussetzungen (Coach & Coachee) und die organisationalen Voraussetzungen (Unternehmen). Dazu gehören auf individueller Ebene beispielsweise Erwartungen und Bereitschaft zur Veränderung sowie auf organisationaler Ebene die Coachingkultur der Organisation (vgl. Abb. 2 und 3).

**Figure 3:**
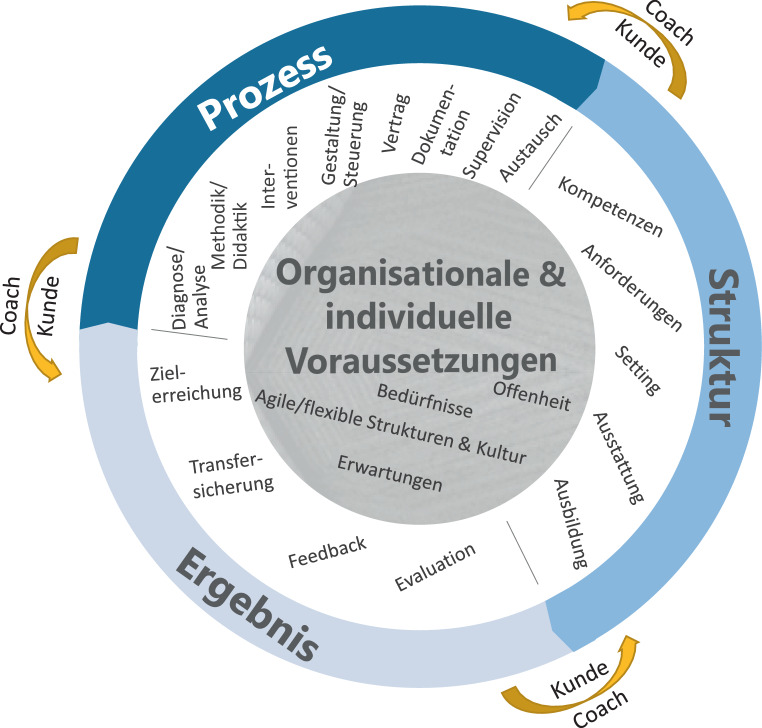

Berninger-Schäfer (2018) hat in Anlehnung an Kühne (2012) und Reindl (2015) eine Sammlung verschiedener Aspekte, die jeweils der Struktur‑, Prozess- und Ergebnisqualität zuzuordnen sind, für Digitalcoaching und Präsenzcoaching aufgestellt. Diese sollen im Folgenden für hybrides Coaching adaptiert und relevanten Kriterien zugeordnet werden. Die Herausforderung des hybriden Ansatzes ist es, jeweils zu entscheiden, welche Kombination von digitalem Setting und Präsenssetting für den Coachee und die jeweilige Coachingsituation Sinn macht. Dies soll an einem Beispiel verdeutlicht werden. Nehmen wir an, in einem Konfliktcoaching arbeitet der Coach mit seinem Coachee mit der Methode der Stuhlarbeit oder systemischen Aufstellung. Dabei wird eine Konfliktsituation aufgearbeitet und der Coachee entwickelt mit Unterstützung des Coaches Lösungsansätze. In der nächsten Coachingsession können die entwickelten Lösungsmöglichkeiten mittels Virtual Reality durchgespielt werden, um ein möglichst „reales Erleben“ zu ermöglichen. Anschließend bekommt der Coachee die Aufgabe, das direkte Konfliktgespräch mit seinem Mitarbeiter:in zu suchen, um das Erarbeitete in der Praxis umzusetzen Dies kann digital vom Coach begleitet werden und in einer weiteren Präsenzsession mit dem Coachee reflektiert und aufgearbeitet werden.

Die folgenden Tab. 1, 2 und 3 zeigen relevante Kriterien zur Struktur‑, Prozess- und Ergebnisqualität, aufbereitet für Präsenz, digital und hybrid.

**Table 1:** 

Strukturqualität			
	Präsenz	Digital	Hybrid
*Setting/Ausstattung*	Räumlichkeiten, Moderationsmaterial, Flipchart, Pinnwand etc	Computer, Notebook, Tablet, Smartphone, Internetverbindung (privat/Firmennetzwerk), digitale Coachingtools, Datenvolumen, VR-Brille	Kombination beider Settings
*Aus- und Weiterbildung*	Coachingmethoden, -interventionen für Präsenzcoaching, kollegiale Beratung, Supervision	Coachingmethoden, -interventionen für digitales Coaching, kollegiale Digitalberatung, Digital-Supervision	Hybrides Weiterbildungsangebot (welche Methoden und Intervention für welches Setting)
*Kompetenzen Coach*	Coachingkompetenz	Coachingkompetenz, Technik- und Medienkompetenz	Coachingkompetenz, Technik- und Medienkompetenz, Flexibilität, Coacheeanpassung, Co-Creation
*Anforderungen Coachee*	Offenheit für Coaching	Offenheit für Coaching, Offenheit für digitale Medien	Flexibilität/Offenheit für hybrides Setting

**Table 2:** 

Prozessqualität			
	Präsenz	Digital	Hybrid
*Diagnostik/Analyse*	Paperbased, im Gespräch	Mittels digitaler Diagnosetools	Kombiniert, z. B. zu Beginn eines Coachings mit digitalem oder analogem Analyse-Canvas
*Methodik/Didaktik*	Entwicklung im Coachinggespräch	Vielfalt an digitaler Coachingtools, strukturierte und standardisierte Coachingprogramme	Kombiniert, was ist geeignet für digital, was für Präsenz
*Interventionen*	Haptisch, greifbar: Körperarbeit, Stuhlarbeit, Entspannungs-übungen, systemische Aufstellung z. B. mit Holzfiguren	Virtual Reality, Avatare zur systemischen Aufstellung	Kombiniert, wann ist welches Setting geeignet
*Prozessgestaltung/-﻿steuerung*	Klassische Coachingprozesse wie z. B. GROW	Wie im Präsenzcoaching übertragbar und durch digitale Tools oder KI abbildbar	Kombiniert, welche Prozessschritte eignen sich für Präsenz, welche für digital oder für eine KI
*Vertragsgestaltung*	Paperbased	Digitale Formulare	Je nach Bedarf/Präferenz des Coachees
*Dokumentation*	Paperbased	Digitale Mitschrift durch Coach und Coachee	Je nach Bedarf/Präferenz des Coachees
*Kollegialer Austausch*	Bei Bedarf	Digital integrierbar, z. B. durch Videozuschaltung	Zwei Kollegen:innen z. B. im Büro und drei Kollegen:innen digital zugeschaltet
*Supervision*	Bei Bedarf	Digital integrierbar, z. B. durch Expertenchat, Videozuschaltung	Gruppe Coaches in Präsenz, Supervisor:in digital zugeschaltet

**Table 3:** 

Ergebnisqualität			
	Präsenz	Digital	Hybrid
*Zielerreichung*	Schriftliche Fixierung z. B. am Flipchart	Digital abgebildet, z. B. mittels Skalen	Kombiniert, digital abgebildet und in Präsenz weitere Ausarbeitung
*Transfersicherung*	Präsenzcoaching nach z. B. zwei Monaten	Digitale Coachingsession z. B. nach zwei Monaten	Kombiniert, z. B. ein bis zwei digitale Sessions und abschließend Präsenzcoaching
*Feedback*	Paperbased oder mündlich nach Coaching oder jeder einzelnen Coachingsession	Digitales Feedbacktool nach jeder Coachingsession und/oder nach dem gesamten Coaching	Kombiniert, z. B. erst digitales Feedback und anschließend gemeinsames Feedbackgespräch
*Evaluation*	Paperbased	Integrierte digitale Evaluationstools	Kombiniert, digitale Evaluationstools und persönliches Interviewgespräch

Die folgenden Abb. 2 und 3 zeigen die qualitativen Gestaltungskriterien für hybrides Coaching im Überblick.

Neben den Qualitätskriterien spielen Ethikrichtlinien im Coaching eine zentrale Rolle (vgl. Berninger-Schäfer 2018). Dies gilt für den Präsenzteil im Coaching als auch für den digitalen Part im Coaching. Die Einhaltung von Ethikrichtlinien sind im Coaching von zentraler Bedeutung und sie gehören zur Selbstverpflichtung professionell arbeitender Coaches (Berninger-Schäfer 2018). Berninger-Schäfer (2018) führt dazu die folgenden zentralen Aspekte für digitales Coaching auf:

- Grundhaltung und Menschenbild: Hierbei sind Wertschätzung, Empathie und respektvoller Umgang unabhängig von Herkunft, Geschlecht, sexueller Orientierung, Hautfarbe, Weltanschauung, nationaler Herkunft, Alter, Religion, Sprache, Kultur, Lebensgestaltung und Status von zentraler Bedeutung.
- Fachliche Kompetenz: Dies impliziert, dass sich das Leistungsangebot auf wissenschaftlich fundierten Konzepten stützt. Das Wissen und Können sollte zudem in nachweisbaren Qualifikationen erworben worden sein.
- Technische Kompetenz: Hier wird vorausgesetzt, dass das Wissen über verschiedene digitale und virtuelle Nutzungsmöglichkeiten im Coaching regelmäßig aktualisiert wird. Auch eine ausreichend technische Ausstattung sollte vorhanden sein. Die Coachees sollten zudem für die Anwendung der digitalen Medien im Coaching vorbereitet und unterstützt werden.
- Transparenz und Integrität: Diese beiden Aspekte bedeuten, dass die Coachees über die Vorgehensweisen, Methoden und Medien sowie deren mögliche Auswirkungen sowie über die Datensicherheit im digitalen Coaching informiert und aufgeklärt werden. Eine wichtige Voraussetzung ist die Zustimmung der Datenschutzerklärung. Der Coach gibt zudem Transparenz über die eigenen Qualifikationen, Mitgliedschaften und Erfahrungen.
- Vertraulichkeit: Unter diesem Punkt fällt die geschützte Datenverwaltung und Datenübertragung wie zum Beispiel Verschlüsselung, Firewalls, Passwörter und Virenschutz. Die Datenschutzbestimmungen hinsichtlich Verschwiegenheit und Datensicherheit sind einzuhalten. Informationen dürfen nur nach ausdrücklicher Genehmigung durch den Coachee weitergegeben werden.

Ein weiterer Aspekt, der hier ergänzt werden sollte, ist die Ermöglichung der Barrierefreiheit durch digitales bzw. hybrides Coaching. Denn digitale Medien können Bildungsangebote an die besonderen Bedürfnisse von Menschen mit Behinderungen anpassen.

Im Folgenden wird zur Veranschaulichung ein Praxisbeispiel eines Coachings mit hybridem Ansatz dargestellt.

## Praxisbeispiel zum Coaching mit hybridem Ansatz

**Figure 4:**
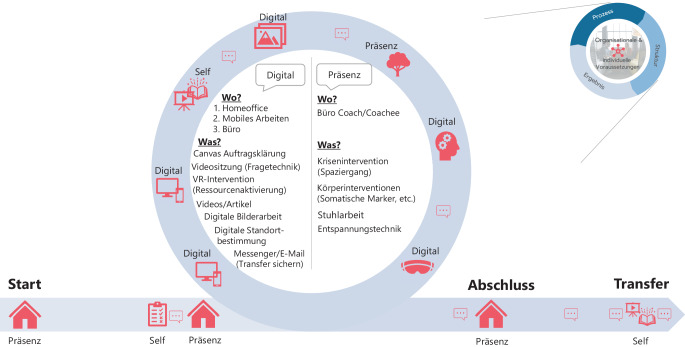

Das Praxisbeispiel bezieht sich auf eine Ende 30-jährige Führungskraft im Vertrieb einer Bank. Die Coachee war zunächst in einer Bankfiliale beschäftigt und wurde zur Regionalleiterin einer Region ernannt. Sie ist in ihrer neuen Rolle viel unterwegs und ihr Team sitzt an mehreren Standorten. Die Aufgabe hat dadurch deutlich an Komplexität gewonnen. Das Ziel des Coachings bezieht sich auf die neuen Rollenanforderungen und die Führung von verteilten Teams. Das Ziel und in dem Falle der „Shift“ wurde spezifisch definiert und auf der Mindset- und Verhaltensebene (heute und in der Zukunft) sowie auf den Impact des Coachings herunter gebrochen. Der Alltag der Coachee ist dabei bestimmt von hohen Wechselwirkungen, die sich auch im Coaching zeigen. Es kann sein der Coachingtermin wurde in Präsenz vereinbart und muss dann kurzfristig in einem digitalen Format umgesetzt werden, da die Coachee an dem Tag nicht mehr vor Ort zur Verfügung steht. Die Anforderungen an vor allem Struktur und besonders Prozess verändern sich damit stark. In dem Beispiel zeigt sich auch die hohe zukünftige Anforderung an Coaches an Design von Coachingmaßnahmen und -Prozessen sowie an den Umgang mit Wechselwirkungen. Die Nutzung unterschiedlicher Medien, Formate oder Methoden bedarf auch einer starken Selbstreflexion beim Coach. Beispielsweise wird in dem Beispiel vor-Ort, in Messenger, E‑Mail und Videoformaten kommuniziert. Dabei ist jedes Mal zu entscheiden, welche Methode nun am Wirksamsten und damit das Coaching effizient und effektiv ist. Die Abb. 4 zeigt exemplarisch den Ablauf mit der Coachee. Zu Beginn des Coachings wurde anhand eines Canvas die Co-Creation gestartet und die Coacheebedürfnisse eruiert. Darin enthalten sind zum Start beispielsweise Strukturmerkmale und Prozessaspekte: Wie soll das Setting sein? Welche digitalen Formate kommen in Frage? Wie ist die IT-Ausstattung? Wie erfolgt die Dokumentation der Coachingmaßnahme? Wer übernimmt welche Aufgabe? Für den Start der Co-Creation haben sich besonders Skalierungsfragen als gut erwiesen. Interessant war in dem Fall, dass bei der Zielbearbeitung ein Zwischenziel integriert werden musste. Die Coachee wollte einen Coachingtermin dazu nutzen sich auf ein digitales Vertriebsmeeting vorzubereiten. Zunächst war der Termin in Präsenz angesetzt, wurde aufgrund des Vertriebsmeetings dann in digital abgehalten. Dazu hat sich das digitale Medium, das auch zu dem Vertriebsmeeting passt, als Coachingformat besonders geeignet.

## Reflexion und Empfehlungen für Forschung und Praxis

Die Arbeitswelt ist in einer fortschreitenden Flexibilisierung und damit in einer hybriden Arbeitsorganisation angekommen. Dies hat sich durch COVID-19 deutlich verstärkt. Coaching muss sich dieser Arbeitswelt anpassen. Der Blick zu Blended-Therapie und Blended-Learning zeigt das Potenzial der Verzahnung von Präsenzcoaching und digitalem Coaching in einem hybriden Coachingansatz. Beide Ansätze zeigen auf, dass die Wirkung einer Verzahnung deutlich höher ist als bei einem einseitigen Einsatz von digital oder Präsenz. Der hybride Coachingansatz ist geprägt durch seine Flexibilität, Vernetzung, Fokussierung, Vielseitigkeit und Individualität. Zudem muss er sich an Qualitätskriterien messen lassen, damit die Qualität im Coaching gegeben ist. Aus der Blended-Therapie abgeleitet lässt sich der integrierte Ansatz der verzahnten Psychotherapie als Grundmodell für die Entwicklung eines hybriden Coachingansatzes nutzen. In der Blended-Therapie wird der integrierte Ansatz zur Flexibilisierung der Psychotherapiemöglichkeiten genutzt. Dies wird nicht explizit mit der Flexibilisierung der Arbeitswelt in Einklang gebracht bzw. begründet, sondern mit der Steigerung der Wirksamkeit und der besseren Ressourcennutzung von Psychotherapie. Daher eignet sich der integrierte Ansatz besonders gut für den hybriden Coachingansatz. Die hohe Komplexität und die damit verbundenen Wechselwirkungen in einem verzahnten hybriden Coachingansatz stellen neue Herausforderungen an Coach und Coachee. Die Coacheebedürfnisse und der Co-Creation Prozess zwischen Coach und Coachee als wesentliche Elemente eines hybriden Coachingformats helfen dabei die hohen Anforderungen zu bewältigen. Sie sind geradezu essentiell und unerlässlich. Für Coaches sind beide Elemente durchaus neu und bedürfen der Vorbereitung und Qualifizierung. Dann gelingt es in Zukunft auch tatsächlich auf Augenhöhe mit dem Coachee zu sein. Die neue Generation der Führungskräfte wird zukünftig und auch heute schon das Element der Co-Creation bereits aus der Lernwelt- und Lernkultur mitbringen und für ganz normal erachten und es damit auch im Coaching einfordern. Das Mindset von Coaches sollte sich daher darauf einstellen und anpassen. Dennoch wird auch zukünftig die Aufgabe beim Coach liegen, die Coachees im Coachingprozess zu „steuern“, Struktur zu geben und den roten Faden zu halten. Im hybriden Coachingansatz wird es zusätzlich wichtig sein, Reflexionsphasen verstärkter einzubauen. Insbesondere, wenn digitale Formate wie KI Coaching integriert werden. Dann ist der reale Coach unabdingbar, dies in den Phasen zu begleiten und quasi eine Qualitätssicherung zu leisten. Qualitätssicherung heißt in dem Kontext die qualitativen Gestaltungskriterien mit dem Coachee regelmäßig zu überprüfen. Eine Art Review und in eine gemeinsame Retrospektive zu gehen.

Die Wirksamkeit von hybridem Coaching gilt es in empirischen Studien in der Zukunft zu untersuchen. Die Vorgehensweise sollte wie folgt aussehen:

1. Ableitung von Szenarien im hybriden Coaching auf Basis des Grundmodells des integrierten Ansatzes und den hier entwickelten qualitativen Gestaltungskriterien im hybriden Coaching
2. Prüfung und Testung der Szenarien mit Probanden:innen
3. Evaluation der Szenarien z. B. auf Basis des Evaluationsmodells von Greif (2016)

Dabei sollten u. a. folgende Forschungsfragen beantwortet werden:

- Welche Voraussetzungen müssen im hybriden Coaching gegeben sein (technisch, organisatorisch, individuell)?
- In welcher Kombination von digitalem Coaching und Präsenzcoaching ist hybrides Coaching am wirksamsten? Wie wird die Wirksamkeit gemessen?
- Was für eine „Dosierung“ von Coaching bei der Verteilung von Präsenz und Digital bietet sich je nach Coacheetypus an?
- Wie können Co-Creation Fähigkeiten für das hybride Coaching aussehen und bei Coach und Coachee entwickelt werden? Wie kann daraus ein guter Designprozess abgeleitet werden?
- Welche Methoden und Interventionen eignen sich für den hybriden Coachingprozess?
- Inwieweit kann hybrides Coaching die Nachhaltigkeit der Coachingergebnisse unterstützen?
- Welche hybriden Szenarien bieten sich für die Coachingpraxis idealerweise an?

Dabei sollte es gelingen, im Grundsatz die Verzahnung und deren Effektivität sowie Wirkung entsprechend auf eine nachweisbare Evidenzlage zu bringen. Genau dies besser zu erforschen und zu verstehen, sollte ein Fokus der Wissenschaftsorientierung im Coaching sein. Aktuell wird sehr stark der Fokus auf digitales Coaching gelegt. Das Evaluationsmodell von Greif (2016) zu verknüpfen mit den qualitativen Gestaltungskriterien im hybriden Coaching unter Berücksichtigung des Grundmodells des integrierten Ansatzes aus der Blended-Therapie ist nach Ansicht der Autoren ein vielversprechender Ansatz für die weitere wissenschaftliche Bearbeitung. Damit sollte es gelingen, den beschriebenen Ansatz auf ein theoretisches Fundament zu bringen und weitere Ableitungen für die Forschungspraxis zu schaffen.

Für die Coachingpraxis bedeutet dies, sich als Coach deutlich mehr mit dem Coachee in den Dialog zu begeben. Dabei sollten folgende Fragen beantwortet werden: Wo stehen meine Coachees und die Organisation hinsichtlich der neuen und digitalen Arbeitswelt? Wie digital ist mein Coachee selbst und welche Fähigkeiten besitzt er, in einen hybriden Coachingprozess zu gehen. Eine Überforderung des Coachees wäre fatal und geradezu hinderlich für das eigentliche Coachingziel. Diese Fragen sollten sich auch die Coaches selbst stellen. Eine praktische Orientierung an den qualitativen Gestaltungskriterien ist hier sicherlich hilfreich. Unterstützend könnte es auch sein, in Peer-Gruppen von Coaches, den Co-Creation Prozess zu üben oder digitale Elemente auszuprobieren. Gerade die neue Generation von Coachees wächst in einer Arbeitswelt auf, die von Design Thinking Prozessen geprägt ist. Darin ist Prototyping und Experimentieren gefordert. Wird dies offen und ehrlich vorab angesprochen und dann ausprobiert, entspricht gerade dies einem Co-Creation Prozess. Die neuen Generationen empfinden genau dies als Dialog auf Augenhöhe und nicht als mangelnde Kompetenz des Coaches.
